# *Helicobacter pylori* infection and inflammatory bowel disease: a 2-sample Mendelian randomization study

**DOI:** 10.3389/fmicb.2024.1384285

**Published:** 2024-10-21

**Authors:** Yurong Cui, Jinxin Li, Bing Zhao, Junying Liu

**Affiliations:** ^1^Department of Digestive Diseases, The First Affiliated Hospital of Henan University of Chinese Medicine, Zhengzhou, China; ^2^The First Clinical Medical College, Henan University of Chinese Medicine, Zhengzhou, China

**Keywords:** Crohn's disease, *Helicobacter pylori*, inflammatory bowel disease, Mendelian randomization, ulcerative colitis

## Abstract

**Introduction:**

Observational studies have discovered a contradictory phenomenon between *Helicobacter pylori (H. pylori)* infection and inflammatory bowel disease (IBD). The study aimed to confirm the causal association between *H. pylori* and IBD, including ulcerative colitis (UC) and Crohn's disease (CD).

**Methods:**

We conducted a Mendelian randomization (MR) study with two sample Genome-Wide Association Studies (GWAS) to determine whether there is a causal relationship between *H. pylori* infection and IBD, as well as the possible pathogenic factors that may be involved. The reliability of the main MR assumptions was examined through a series of sensitivity analyses.

**Results:**

Two genetic variants (SNPs) previously identified were employed as instrumental variables (IVs) for *H. pylori* infection. GWAS data for IBD, UC, and CD were obtained from the recent DF10 release10 of the FinnGen study. Our findings indicated a significant association between *H. pylori* seropositivity and an increased risk of IBD and UC (IBD: OR: 1.16, 95% CI, 1.03–1.31, *P* < 0.05; UC: OR: 1.22, 95% CI, 1.08–1.37, *P* < 0.001) while no causal relationship with CD (*P* > 0.05). Analysis of the main virulence pathogenic factors revealed a causal relationship between cytotoxin-associated protein A (CagA) and IBD and UC (IBD: OR: 1. 06, 95% CI, 1.001–1.11, *P* < 0.05; UC: OR: 1.07, 95% CI, 1.004–1.14, *P* < 0.05), while no correlation was found for vacuolar cytotoxin A (VacA) (*P* > 0.05). After applying the False Discovery Rate (FDR) correction, the causal relationship between CagA and the risk of IBD or UC was no longer statistically significant.

**Conclusion:**

This study suggests a potential causal relationship between H. pylori infection and IBD, particularly UC. The effect may be more pronounced in individuals with previous *H. pylori* infections.

## Introduction

Inflammatory bowel disease (IBD) is a chronic inflammatory disorder of the gastrointestinal tract, clinically encompassing conditions such as Crohn's disease (CD) and ulcerative colitis (UC) (Bruner et al., 2023). IBD represents a globally prevalent chronic condition significantly impacting patients' quality of life. Epidemiological data indicate a rapid increase in incidence rates in emerging nations with historically low prevalence, while rates remain stable or are rapidly rising in Western countries (Kaplan and Windsor, 2021). The pathogenesis of IBD is thought to involve inappropriate and sustained inflammatory responses to commensal microorganisms in genetically predisposed individuals, although the precise mechanisms remain unclear (Khor et al., 2011).

*Helicobacter pylori (H. pylori)* infection is highly prevalent, affecting ~60% of the global population (Hooi et al., 2017). *H. pylori* is a major risk factor for the development of gastric cancer, and recent research has increasingly implicated *H. pylori* in the pathogenesis of other diseases. Cytotoxin-associated antigen A (CagA) and vacuolar cytotoxin A (VacA) are among the important virulence factors associated with *H. pylori* (Nejati et al., 2018).

Current evidence suggested that *H. pylori* infection may induce exacerbation of inflammatory responses, potentially contributing to the pathogenesis of IBD. However, some argued that *H. pylori* infection may have a protective effect against IBD. This controversial topic has led scholars worldwide to conduct numerous clinical studies, yet the clinical data remain contradictory, lacking consensus (Papamichael et al., 2014). Mendelian randomization (MR), which uses genetic variations to investigate causal relationships between exposures and outcomes, has emerged as a powerful tool to address the limitations and confounding factors present in observational studies (Emdin et al., 2017; Cai et al., 2023). This study applied MR analysis to elucidate causal relationships and guide further research into the pathogenesis of IBD.

## Methods

### Mendelian randomization design

**Figure 1:**
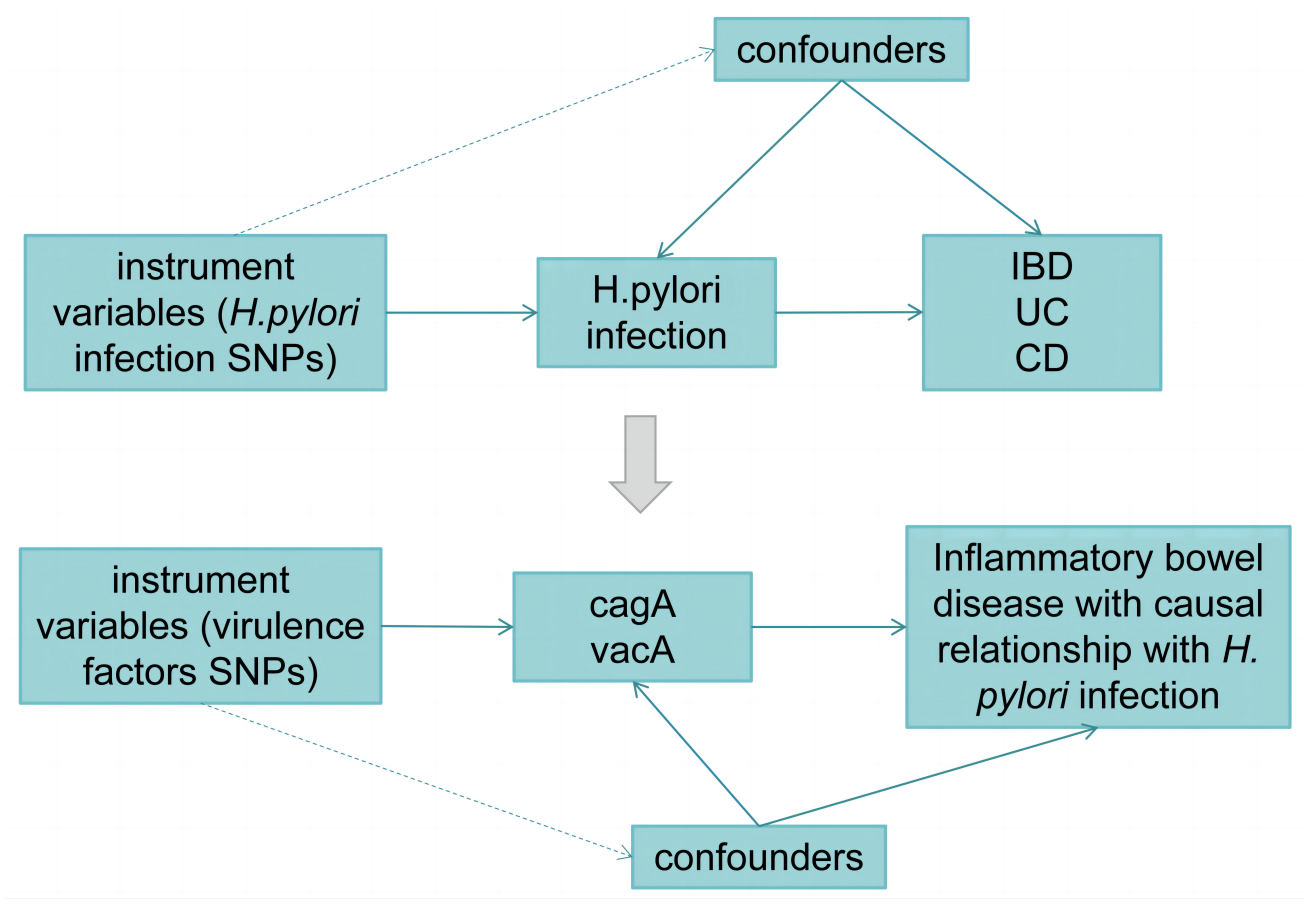
Workflow of the MR study.

Figure 1 illustrates the workflow of the current Mendelian randomization (MR) study. In our study, we utilized genetic variants as instrumental variables (IVs) for MR analysis. The validity of our MR study relies on three core assumptions:

(1) The relevance assumption: genetic variants are closely associated with the exposure.

(2) The independence assumption: genetic variants are unrelated to any confounding factors that may mediate the pathway from exposure to outcome.

(3) The exclusion-restriction assumption: genetic variants affect the outcome only through the exposure.

The effectiveness of our MR analysis hinges upon these fundamental assumptions.

### Data sources

Genetic instrumental variables (IVs) can be obtained through two main approaches: from previous literature or directly from summary statistics of Genome-Wide Association Studies (GWAS). The genetic IVs for *Helicobacter pylori* infection were abtained from a prior study by Mayerle et al. (2013). Urea breath test, *H. pylori* fecal antigen detection, and serological testing are commonly used diagnostic methods for *Helicobacter pylori* in clinical practice. This study utilized the detection of anti-*H. pylori* IgG titers to determine serum positivity, which served as an indicator of current or past infections. A positive fecal *H. pylori* antigen detection was considered indicative of an active infection. The study cohort consisted of 10,938 participants, all of European descent. Two single nucleotide polymorphisms (SNPs), rs10004195 (*P* = 1.4 × 10^−8^) and rs368433 (*P* = 2.1 × 10^−8^, were identified as the strongest genetic variants associated with H. pylori seropositivity, with both SNPs exhibiting *P*-values below the genome-wide significance threshold of 5 × 10^−8^. IVs related to CagA were obtained from publicly available data collected from the European Bioinformatics Institute (EBI) database (https://gwas.mrcieu.ac.uk/datasets/ebi-a-GCST90006911/), comprising 985 samples with 9,165,056 SNPs, among which 15 SNPs with a *P-*value < 5 × 10^−6^ were considered associated with CagA. IVs related to VacA were also acquired from the EBI database (https://gwas.mrcieu.ac.uk/datasets/ebi-a-GCST90006916/), consisting of 1,571 samples with 9,178,635 SNPs, where 15 SNPs with a *P-*value < 5 × 10^−6^ were deemed associated with VacA.

A linkage disequilibrium (LD) clumping algorithm with an R^2^ threshold < 0.001, window size = 10,000 kb, and a significance threshold of *P* < 5 × 10^−8^ was utilized to remove SNPs in strong LD. Subsequently, to ensure that the effect alleles belonged to the same allele, a harmonization of exposure and outcome datasets was performed to eliminate SNPs with intermediate allele frequencies and SNPs with ambiguous alleles. Following these stringent selection criteria, these SNPs were used as IVs for subsequent analysis.

GWAS data for IBD, UC, and CD were obtained from the recent DF10 release (Kurki et al., 2023) of the FinnGen study (https://www.finngen.fi/en), with cases strictly defined through rigorous screening. Details of the GWAS included in this study are provided in Table 1.

**Table 1:** Details of the GWAS.

**Phenotype**	**Institution or author**	**Population**	**Sample size**	**Year**	**Number of SNPs**	**Web source**
CagA	Butler-Laporte G	European	985 participants	2020	9,165,056	https://gwas.mrcieu.ac.uk/datasets/ebi-a-GCST90006911/
VacA	Butler-Laporte G	European	1,571 participants	2020	9,178,635	https://gwas.mrcieu.ac.uk/datasets/ebi-a-GCST90006916/
IBD	FinnGen	European	9,083 cases/403,098 controls	2022	21,306,349	https://storage.googleapis.com/finngen-public-data-r10/summary_stats/finngen_R10_K11_IBD_STRICT.gz
UC	FinnGen	European	5,931 cases/405,386 controls	2022	21,306,338	https://storage.googleapis.com/finngen-public-data-r10/summary_stats/finngen_R10_K11_UC_STRICT2.gz
CD	FinnGen	European	2,191 cases/392,974 controls	2022	21,305,992	https://storage.googleapis.com/finngen-public-data-r10/summary_stats/finngen_R10_CHRONNAS.gz

### Statistical analyses

The inverse-variance weighted (IVW) meta-analysis with multiplicative random model was used as the major analysis for causal estimation. Cochran's *Q*-test was performed to detect heterogeneity among the genetic variants. The MR–Egger regression model and leave-one-out test were performed as sensitivity analyses and to examine the existence of horizontal pleiotropy that violated the main MR assumptions. MR–Egger regression were applied to detect and correct for pleiotropic effects (Verbanck et al., 2018).

Analyses were performed in R (v.4.3.2) statistical software. The two-sample univariable analyses were performed using the package “TwoSampleMR” (0.5.8). The *P-*values in this study were 2-sided, and values < 0.05 were deemed significant.

## Results

### Causal effect of *H. pylori* infection on IBD, UC, and CD

**Figure 2:**
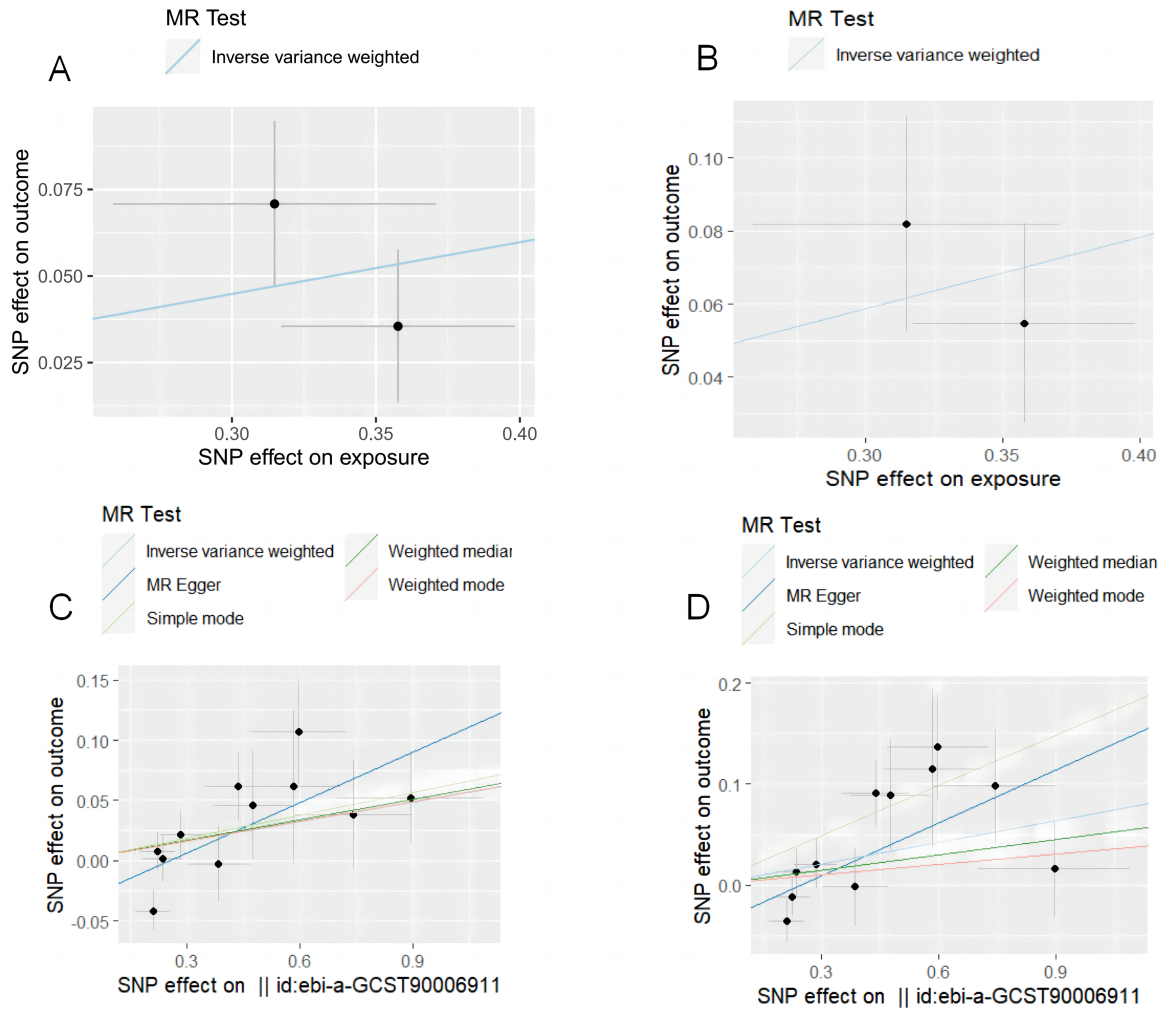
MR test plot. **(A)** *H. pylori* infection on IBD. **(B)** *H. pylori* infection on UC. **(C)** CagA on IBD. **(D)** CagA on UC.

After setting the independence of the exposed data through clumping, and merging the exposure and outcome datasets, the SNP rs10004195 (T > A) and the SNP rs368433 (T > C) were used in the MR analysis working as IVs. The F statistics were were both above 20. All genetic associations were aligned to the allele that increases the *H. pylori* seropositivity. Genetically predicted *H. pylori* infection showed causally association with an increased risk of IBD in the FinnGen GWAS under the IVW method [odds ratio (OR): 1.16, *P* < 0.05; 95% confidence interval (CI), 1.03–1.31; value of *P* = 0.015]. And causally association with an increased risk of UC in the FinnGen GWAS under the IVW method (OR: 1.22, 95% CI, 1.08–1.37, *P* = 0.00090) (Figures 2A, B). While no causal relationship showed on CD (*P* = 0.96).


$$\begin{array}{l} F=\;\left(N-K-1\right)/K^{*}R^{2}/\left(1-R^{2}\right) \end{array}$$

### Causal effect of virulence pathogenic factors of *H. pylori* infection on IBD and UC

After setting the independence of the exposed data through clumping, and merging the exposure and outcome datasets, merging the exposure and outcome datasets, 11 IVs were used for the analysis of CagA, and 14 IVs were used for the analysis of VacA. The F statistics of the selected variables were all above 20, suggesting that there was no strong evidence for weak instrument bias (details of the IVs included in this study are provided in Table 2). Genetically predicted CagA showed causally association with an increased risk of IBD and UC under the IVW method (OR: 1.16, *P* < 0.05; 95% CI, 1.03–1.31; value of *P* = 0.015). And causally association with an increased risk of UC in the FinnGen GWAS under the IVW method (IBD: OR: 1. 06, 95% CI, 1.001–1.11, *P* = 0.042; UC: OR: 1.07, 95% CI, 1.004–1.14, *P* = 0.039) (Figures 2C, D). While VacA showed no causal relationship on IBD and UC (IBD: *P* = 0.65; UC: *P* = 0.51).

**Table 2:** IVs of virulence pathogenic factors.

**Exposure**	**SNPs**	**Beta**	**SE**	**EAF**	***P*-value**	***R* ^2^**	***F*-statistic**
*H. pylori*	rs10004195	0.3576744	0.04048331	0.25	1.40E-18	0.007085939	78.04485335
	rs368433	0.3148107	0.05609599	0.16	2.10E-08	0.002871103	31.48878609
CagA	rs116421363	−0.223345	0.0475812	0.363202	2.68E-06	0.021879521	21.98867066
	rs75170215	0.743256	0.156416	0.02057	2.02E-06	0.022409655	22.53366242
	rs75740599	0.235842	0.0512963	0.252886	4.27E-06	0.021009346	21.09538736
	rs3998182	0.284762	0.0548049	0.27555	2.04E-07	0.026677541	26.94279068
	rs6530847	−0.210266	0.0452734	0.391211	3.41E-06	0.021429294	21.52628924
	rs4268452	−0.384976	0.0840329	0.071203	4.62E-06	0.020862957	20.94526676
	rs117827497	−0.583731	0.125147	0.030462	3.10E-06	0.021610274	21.7121043
	rs117537486	0.596789	0.129736	0.03072	4.22E-06	0.021030687	21.11727615
	rs149747348	−0.895407	0.194003	0.013186	3.92E-06	0.021168772	21.25892825
	rs11858369	0.437721	0.0864978	0.068381	4.18E-07	0.025339712	25.55653255
	rs118006294	−0.475295	0.101815	0.050367	3.04E-06	0.021645273	21.74804622
VacA	rs113063793	0.373402	0.075648	0.056242	7.97E-07	0.015272089	24.33353121
	rs72645538	0.752792	0.164377	0.011992	4.66E-06	0.013174438	20.94665351
	rs113845906	0.413478	0.0845187	0.045066	9.97E-07	0.015005716	23.90264467
	rs1530121	−0.266111	0.0511414	0.86313	1.96E-07	0.016942717	27.04127639
	rs372744619	0.887411	0.173802	0.010519	3.29E-07	0.016323607	26.03675369
	rs77497849	0.264227	0.0538191	0.119223	9.13E-07	0.01511098	24.07289294
	rs7019543	0.18107	0.0380994	0.320078	2.01E-06	0.014173626	22.55815046
	rs59104649	0.68347	0.147207	0.014016	3.44E-06	0.013535899	21.52924239
	rs117077218	0.640222	0.138755	0.016656	3.95E-06	0.013370323	21.26232061
	rs148556020	0.63085	0.137457	0.016883	4.44E-06	0.013229952	21.03610118
	rs11044935	0.428278	0.0905091	0.039367	2.22E-06	0.014052214	22.36216242
	rs73500239	0.236088	0.0500401	0.144805	2.38E-06	0.013970921	22.23096152
	rs9606224	0.507461	0.10223	0.029841	6.91E-07	0.015442357	24.60908004
	rs133537	−0.173609	0.0367088	0.615079	2.25E-06	0.014037453	22.3383378

After all analyses were completed, FDR (False Discovery Rate) correction was applied to the *P*-values of the same disease to reduce the probability of false positives, and ultimately the causal relationship between CagA and the risk of IBD or UC was no longer significant (Table 3).

**Table 3:** IVW and sensitivity analyses.

**Exposure**	**Outcome**	**Number of SNPs**	**IVW**		**FDR**	**Cochran's *Q*-test**	**MR-Egger pleiotropy test**
			**OR (95% Cl)**	***P***	**Adjust** ***P***	***P***	***P***
*H. pylori*	IBD	2	1.16 (1.03–1.31)	0.015^*^	0.0457^*^	0.20	–
	UC	2	1.22 (1.08–1.37)	0.0009^**^	0.0027^**^	0.37	–
	CD	2	1.00 (0.83–1.22)	0.9606	0.9606	–	–
CagA	IBD	11	1.06 (1.001–1.11)	0.0419^*^	0.0629	0.10	0.08
	UC	11	1.07 (1.004–1.14)	0.039^*^	0.0587	0.051	0.12
VacA	IBD	14	1.01 (0.95–1.08)	0.6487	0.6487	–	–
	UC	14	1.03 (0.95–1.11)	0.5109	0.5109	–	–

^*^P < 0.05; ^**^P < 0.01.

### Sensitivity analyses

**Figure 3:**
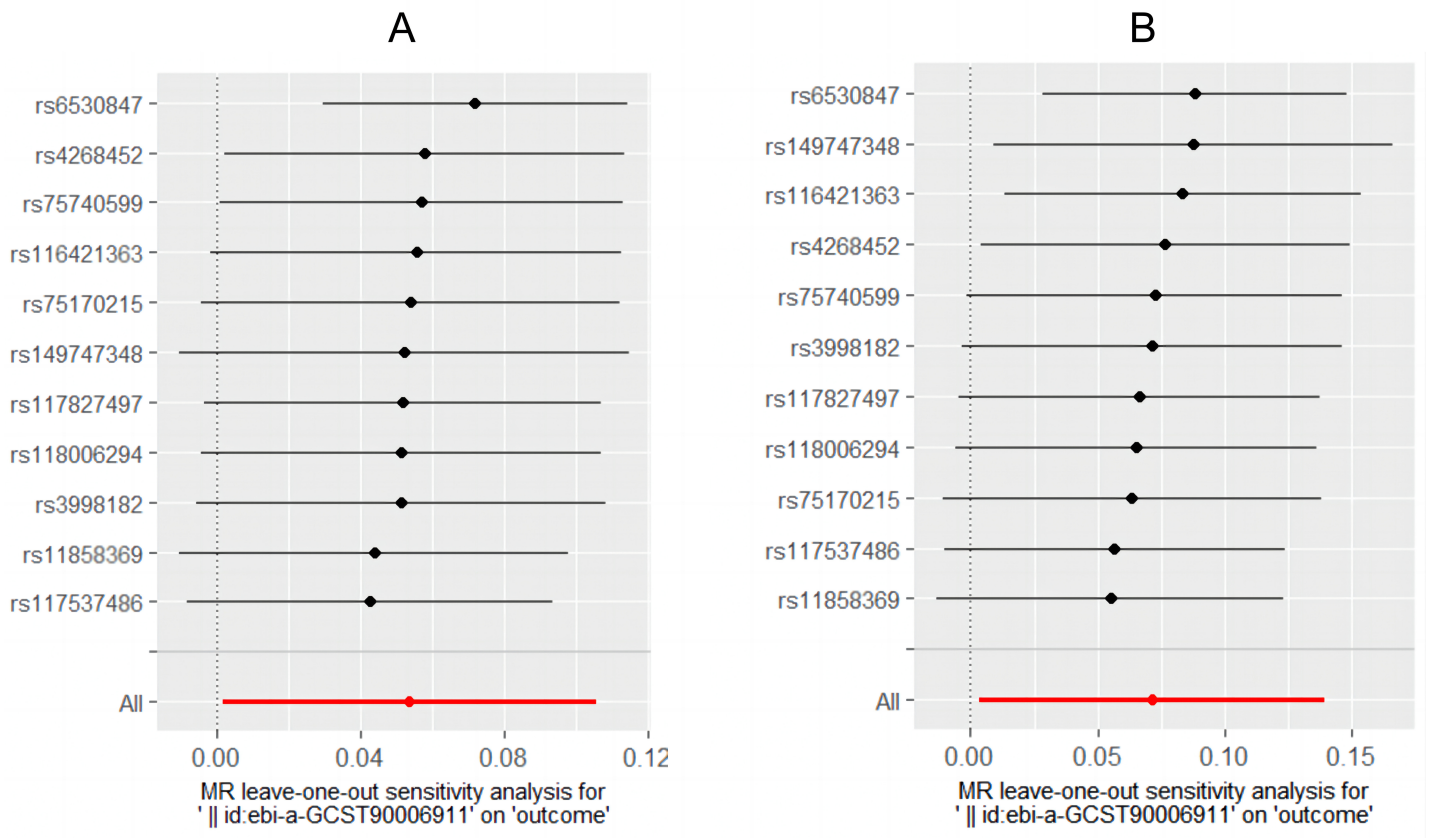
Leave-one-out plot. **(A)** CagA on IBD. **(B)** CagA on UC.

Cochran's *Q-*test showed that the main estimates of *H. pylori* infection and CagA on IBD and UC were hardly affected by any heterogeneity (*P* > 0.05). MR Egger regression model and leave-one-out analyses all showed that the main estimates of CagA on IBD and UC were hardly affected by any pleiotropy (*P* > 0.05) (Table 3). The leaveoneout plot was used to visualize the sensitivity (Figure 3).

## Discussion

In this study, we found an association between *H. pylori* infection and an increased risk of IBD, primarily linked to the risk of UC. Currently, the association between *H. pylori* infection and the risk of IBD diseases has been extensively studied, with some indicating that *H. pylori* infection may induce exacerbation of inflammatory responses, potentially being related to the pathogenesis of IBD.

Due to its microaerobic metabolism, helical shape, and unique motility, *H. pylori* easily colonizes the surface of the gastrointestinal tract (Sonnenberg, 2013). In inflammatory bowel disease, dysregulation of the host's immune response to commensal bacteria is considered a significant potential pathogenic mechanism. When the gastric mucosa mounts an immune response to *H. pylori*, products of the local immune reaction may disseminate to extragastric sites. In animal models, enterohepatic Helicobacter species such as *H. hepaticus* and *H. bilis* have been shown to induce sustained inflammation in the colon and cecum of immunodeficient rodents (Kullberg et al., 1998; Shomer et al., 1997). Some studies also suggested that infection may exert a protective effect on IBD by altering the diversity of the intestinal microbiota, subsequently modifying the local chemical characteristics of the intestine and the pattern of intestinal immune response (Feilstrecker Balani et al., 2023).

Regarding this controversial topic, scholars from various countries have conducted numerous clinical studies, yet their clinical data remain contradictory, lacking unified results. In a study involving 160 Chinese IBD patients (10%) and 80 control subjects (6.3%), there was no significant difference in the detection rate of Helicobacter genus in intestinal biopsy specimens (Zhang et al., 2011). Some studies had found a lower prevalence of *H. pylori* infection in IBD patients compared to control groups, suggesting a protective effect of *H. pylori* infection on the development of IBD (Jin et al., 2013). However, In one study, the PCR positivity rate for Helicobacter genus in UC patients was significantly higher compared to the control group (Thomson et al., 2011). Another study found that the *H. pylori* genus in the intestinal mucosa of CD patients was significantly higher than that in patients without inflammatory bowel disease through PCR (Oliveira et al., 2006). Clinical studies on *H. pylori* infection are inherently observational and lack randomization, prospective design, and blinding, leading to significant limitations and confounding factors such as differences in patient medication history and detection methods. Given this contradictory phenomenon, our study aimed to elucidate the potential causal relationship between *H. pylori* and IBD, the results of MR showed a causal relationship between *H. pylori* infection and increased risk of UC, but no causal relationship with CD. It is worth mentioning that, given the genetic IV sources of *H. pylori* infections included in this MR study, *H. pylori* infections here include both current and past infections. However, the current clinical trials have mainly focused on current *H. pylori* infections, which may also be the reason for the differences in results.

This study further explored the pathogenic virulence factors and identified an association between CagA and an increased risk of IBD, while VacA was not found to have a related effect. However, after FDR correction, there was no causal relationship between CagA and IBD (*P* = 0.0629) or between CagA and UC (*P* = 0.0587). Therefore, the correlation between *H. pylori* infection and increased risk of IBD may not be related to virulence factors. At the same time, two antibodies related to virulence factors, *H. pylori* VacA antibody and *H. pylori* CagA antibody, also represent some current *H. pylori* infections.

To assess the reliability of our study results, we conducted a series of sensitivity analyses. Cochran's *Q*-test, MR Egger, and leave-one-out analyses were employed, yielding robust results. These findings suggest that patients with *H. pylori* infection and associated intestinal symptoms may require screening for IBD. Furthermore, they provide insights for further exploration into the pathogenesis of UC. Perhaps further clinical or laboratory studies are needed to compare current *H. pylori* infections with previous infections. Further research is needed on the pathogenic factors involved. Meanwhile, it once again indicates the possible differences in the pathogenesis between UC and CD.

Although UC and CD have historically been studied together because they share common features, it is clear that they represent two distinct pathophysiological entities (de Souza and Fiocchi, 2016). Widespread activation of humoral immune response was observed in both diseases, resulting in various changes in immunoglobulin subclasses (MacDermott et al., 1989; Scott et al., 1986). A reported human epithelial colon self antigen recognized by tissue bound IgG antibodies in UC has not been recognized by CD mucosa (Takahashi and Das, 1985). This self antigen also exists in typical sites of extraintestinal manifestations of UC, suggesting that antibody mediated immune responses may be related to the intestinal and extraintestinal pathology of UC patients (Halstensen et al., 1993).

In addition, we found that a Mendelian randomization study on the same topic was published in February of this year (Yang et al., 2024), drawing a conclusion of no causal relationship. After comparing with the content of this article, the main reason for the different conclusions drawn is the different sources of *H. pylori* infection exposure data. Our genetic IVs were derived from a study in *JAMA* that identified genetic loci associated with susceptibility to *H. pylori* (Mayerle et al., 2013). Its diagnosis comes from serum anti Helicobacter pylori IgG and *H. pylori* antigen in feces, including current and previous infections. The final determined SNPs have *P*-values < 5 × 10^−8^. The *H. pylori* related genetic IVs obtained by Yang et al. were sourced from the EBI database, which includes Anti-*H. pylori* IgG Phenotype data from previous infections. There were no SNPs in this database with *P*-values < 5 × 10^−8^. When the conditions were relaxed to *P*-values < 5 × 10^−6^, 11 SNPs were selected for analysis. Therefore, this may not conflict with our research findings.

The current clinical research mainly focused on grouping analysis based on whether *H. pylori* is currently infected, and inconsistent results have been obtained from each group. In this MR analysis, after FDR correction, it was found that there is no causal relationship between virulence factor related IVs and IBD in some current infections, while genetic IVs determined based on current and past *H. pylori* infections have a causal relationship with IBD, especially UC. Currently, some studies had reported a suspected increase in the probability of IBD after *H. pylori* sterilization (Homolak et al., 2021; Chiba et al., 2016). A retrospective cohort study evaluated more than 5 million patients receiving *H. pylori* eradication treatment, and found that the incidence of IBD increased, and the incidence rate of UC in patients ≥ 30 years old increased more significantly (Mizukami et al., 2023). Therefore, based on current clinical reports and MR results, we speculate that a history of *H. pylori* infection is associated with an increased risk of IBD (especially UC). The reason for the increase in the incidence rate of IBD after *H. pylori* eradication may include factors of past *H. pylori* infection (antibody mediated immune response, changes in flora, etc.) in addition to antibiotics. However, this speculation cannot be validated through MR analysis and may require further clinical or laboratory research.

## Conclusion

Our study suggests a potential causal relationship between *H. pylori* infection and IBD, especially UC. The effect may be more pronounced in individuals with previous *H. pylori* infections. Further research is needed on the pathogenesis of IBD.

## Data Availability

The original contributions presented in the study are included in the article/supplementary material, further inquiries can be directed to the corresponding author.
